# Revisiting the relationship between mercury emission and bioaccumulation

**DOI:** 10.1016/j.eehl.2022.12.001

**Published:** 2023-01-07

**Authors:** Martin Tsz-Ki Tsui, Sae Yun Kwon, Mi-Ling Li, Kevin Bishop

**Affiliations:** a School of Life Sciences, Earth and Environmental Sciences Programme, Institute of Environment, Energy and Sustainability, The Chinese University of Hong Kong, Shatin, N.T., Hong Kong SAR, China; b State Key Laboratory of Marine Pollution, City University of Hong Kong, Kowloon Tong, Kowloon, Hong Kong SAR, China; c Division of Environmental Science and Engineering, Pohang University of Science and Technology, 77 Cheongam-Ro, Nam-Gu, Pohang 37673, South Korea; d School of Marine Science and Policy, University of Delaware, 306 Robinson Hall, Newark, DE 19716, United States; e Department of Aquatic Sciences and Assessment, Swedish University of Agricultural Sciences, Uppsala SE, 75007, Sweden

Atmospheric emission and deposition of mercury (Hg), a toxic metal of global concern, has been long considered to control environmental Hg levels in water, soil, sediment, and ultimately fish, which is the major exposure source for humans. In the last two decades, a number of studies of varying spatiotemporal scales and approaches have shown that fish Hg, particularly in lakes and open oceans, is positively and linearly correlated with atmospheric Hg deposition through both dry and/or wet deposition [[1], [2], [3]]. These studies were mainly conducted in North America and Europe. However, little is known about the relationship between atmospheric Hg and fish Hg in other continents and/or ecosystem types (i.e., rivers and coastal zones).

In East Asia, Hg emission has been rising steadily over the last few decades until around 2013 [4], and a recent study by Zhang et al. [5] examined the extensive fish Hg database (35,464 individual fish data) in China between 1980 and 2014. The wild and farmed fish samples were collected from a variety of environments ranging from freshwater to marine habitats and from relatively pristine to highly Hg-contaminated environments. Data of potentially poor quality, especially due to earlier analytical instruments and methods, have been removed to ensure accurate spatiotemporal comparison across China.

Interestingly, Zhang et al. [5] discovered that, while Hg emission from China had been increasing by 70 Mg per five years since the economic reform in the early 1980s, fish Hg levels declined measurably by an average of 0.024 mg/kg wet wt. per five years. The authors attribute this to the cascades of ecological modification, such as the shortened food chain length and suppression of Hg biomagnification, resulting from the increased demand for fish consumption driven by population growth and/or dietary preference. Obviously, the observed Hg trend under the substantial socioeconomic pressure in this large East Asian country is very different from those reported in North America and Europe.

While it appears clear from the paper that Hg emission and fish Hg levels are inversely related to one another over the period of 35 years [5], we suggest examining the observed trend at a finer resolution and considering multiple factors. Specifically, in addition to the socioeconomic influence on fish consumption, we recommend taking other possible explanations into consideration when assessing the temporal trends in fish Hg. Previous studies and review papers, which have discussed possible environmental, landscape, and ecological factors driving fish Hg level [[6], [7], [8]], provide a number of hypotheses that could be examined in the context of the Chinese records assembled by Zhang et al. [5]. We believe that this would be of particular value for evaluating the effectiveness of the Minamata Convention on Mercury in the future.

Among these hypotheses, we suggest considering the possibility of a substantial temporal delay from atmospheric Hg deposition to the bioaccumulation of Hg in fish. Most of the atmospheric Hg deposition would be in the form of inorganic Hg (e.g., gaseous elemental Hg and reactive Hg(II)). Having a larger pool of inorganic Hg in the environment would theoretically provide more substrate (i.e., bioavailable Hg(II)) for producing the highly bioaccumulative methylmercury (MeHg). Depending on the watershed-to-lake area ratio, however, it is possible that some of the deposited Hg(II) may be sequestered into terrestrial surfaces, located adjacent to aquatic water bodies (littoral, riparian, or coastal regions), and transformed into MeHg with a substantial temporal delay [9].

A second hypothesis, as suggested by Zhang et al. [5], is that trophic position and fish age are crucial factors controlling MeHg concentration in fish as they are associated with the extent of MeHg biomagnification and exposure time, respectively. The high demand for both freshwater and coastal fish by the large population in China probably has driven the destruction of many aquatic food webs, which, in turn, shortened the food chain length as well as downsized fish size (since larger fish was quickly removed for human consumption). Smaller fish and fish at lower trophic levels (e.g., herbivores or planktivores) play an increasingly important role in the Chinese fish market and can be largely responsible for the lower MeHg levels as observed by Zhang et al. [5].

Third, as Zhang et al. [5] suggested, the severe eutrophication and algal bloom in both inland and coastal environments would stimulate faster growth of aquatic biota, which may, in turn, dilute the MeHg levels in the prey and the predatory fish. However, a recent study by Lei et al. [10] showed significant methanogen-mediated MeHg production in eutrophic lakes via algal organic matter. The relative importance of MeHg biodilution and production in eutrophic water bodies is unclear and requires further investigation in the future.

Fourth, the environmental/landscape factors leading to MeHg production or enhanced Hg bioavailability would also influence the spatial variability in fish Hg levels. Such a notion is particularly evident from the series of sediment-fish Hg monitoring data published by the US Geological Survey in the western US [[11], [12]]. In these studies, it was found that spatial variability in fish Hg is dictated by both the landscape structure, which controls the external Hg input, and biome-type (i.e., wetland, temperate forest, etc.), which mediates MeHg production and bioavailability. With the large-scale economic reform and land use changes, China has been facing widespread deforestation and wetland drainage [13,14], which may lead to reduced dry deposition of Hg onto tree canopies and more oxidative environments which may not favor microbial Hg methylation. These may partially contribute to the decreasing trend of Hg in fish observed by Zhang et al. [5].

Zhang et al. [5] also reported that inland fish (e.g., Tibet and Xinjiang) tended to have higher Hg than those at the southeastern coastal sites in China. While the much lower extent of fisheries catches for human consumption in the inland region was raised as a possible explanation for the higher fish Hg levels, it is also possible that there is more Hg methylation in freshwater sediment and water columns [15] compared to the more rapidly flowing waters of coastal water systems. Thus, we suggest that a more detailed analysis of the habitat-specific fish Hg and/or aqueous and sedimentary MeHg trends in China is warranted for a better understanding of the long-term trend of fish Hg under rising Hg emission.

Therefore, given the temporal changes in fisheries composition (e.g., aquaculture *vs.* wild catch, carnivore *vs.* omnivore *vs.* herbivore) and complex socioeconomic and environmental changes over the past 30 years in China, the observed fish Hg trend cannot be easily interpreted on a single, national level. To disentangle how the increasing anthropogenic Hg emissions affect fish Hg burden in China, we suggest selecting ambient sediments and certain fish species, preferably at lower trophic levels to avoid complicated predation relationships for a sustained monitoring program of MeHg/total Hg concentrations along with ecological and environmental factors (e.g., fish age and size, trophic position, and water quality parameters).

We should recognize that all these factors, which happen simultaneously in China and perhaps other rapidly developing countries, may work somewhat differently from those of relatively “more stable” habitat types and fishery compositions in North America and Europe, where atmospheric Hg deposition change is one of the largest factors in play [16]. Since other issues (e.g., the varying fish species, changing habitat type over time, and contamination status of each site, etc.) would complicate the data interpretation in this large-scale study, we urge that monitoring is complemented by experimental approaches such as METAALICUS [2] and SPRUCE [17] that would be helpful in teasing apart the various factors influencing fish Hg data in China and other countries around the world. Looking into the next few decades, an effective evaluation of the Minamata Convention on Mercury would be very useful in China and many other countries in order to better evaluate the benefits of the agreed reduction of Hg emissions by the global community.

## Declaration of competing interest

The authors declare that they have no known competing financial interests or personal relationships that could have appeared to influence the work reported in this paper.
